# A Neural Network-Based Optimal Spatial Filter Design Method for Motor Imagery Classification

**DOI:** 10.1371/journal.pone.0125039

**Published:** 2015-05-01

**Authors:** Ayhan Yuksel, Tamer Olmez

**Affiliations:** Department of Electronics and Communication Engineering, Istanbul Technical University, Istanbul, Turkey; Univerity of Rome Tor Vergata, ITALY

## Abstract

In this study, a novel spatial filter design method is introduced. Spatial filtering is an important processing step for feature extraction in motor imagery-based brain-computer interfaces. This paper introduces a new motor imagery signal classification method combined with spatial filter optimization. We simultaneously train the spatial filter and the classifier using a neural network approach. The proposed spatial filter network (SFN) is composed of two layers: a spatial filtering layer and a classifier layer. These two layers are linked to each other with non-linear mapping functions. The proposed method addresses two shortcomings of the common spatial patterns (CSP) algorithm. First, CSP aims to maximize the between-classes variance while ignoring the minimization of within-classes variances. Consequently, the features obtained using the CSP method may have large within-classes variances. Second, the maximizing optimization function of CSP increases the classification accuracy indirectly because an independent classifier is used after the CSP method. With SFN, we aimed to maximize the between-classes variance while minimizing within-classes variances and simultaneously optimizing the spatial filter and the classifier. To classify motor imagery EEG signals, we modified the well-known feed-forward structure and derived forward and backward equations that correspond to the proposed structure. We tested our algorithm on simple toy data. Then, we compared the SFN with conventional CSP and its multi-class version, called one-versus-rest CSP, on two data sets from BCI competition III. The evaluation results demonstrate that SFN is a good alternative for classifying motor imagery EEG signals with increased classification accuracy.

## Introduction

A Brain-Computer Interface (BCI) is an alternative method of communication between a user and system in which the user does not need to use his/her brain-muscular pathways to control an external device [1]. Because it is a direct communication method with the brain and outer world, the BCI system emerges as a useful communication and control method for severely paralyzed people. In such a system, the user should generate different signal patterns with his brain for different commands. Moreover, discriminating these brain patterns (typically EEG) and translating them to control commands for an electronic device is the most important part of the BCI system. In Motor Imagery (MI)-based BCI systems, the kinaesthetic imagination of body movement results in oscillations called event-related synchronization/desynchronization in the sensorimotor cortex in the *μ* and *β* frequency bands [2, 3].

Due to the topographical organization in the motor cortex, different motor imagery tasks can be identified based on their specific spatial location of related ERD rhythms [4]. However, due to the volume conduction effect, scalp EEG signals recorded from a specific area involve a mixture of several cortical sources located in different areas. Thus, raw scalp EEG potentials have poor spatial resolution [4]. To eliminate the volume conduction effect and reach the actual underlying signal sources, a spatial filtering step is an indispensable technique [5].

Common spatial patterns (CSP) is a very popular and powerful spatial filtering method used in motor imagery EEG classification [6]. When using band power features, CSP computes spatial filters, aiming to obtain optimal discrimination between two classes [7]. CSP finds optimal spatial filters that maximize the ratio of average variances that belong to two different classes. Computationally, CSP is solved by simultaneously diagonalizing the two covariance matrices of the two classes [8]. A computed CSP spatial filter projects the multi-dimensional EEG time domain signal to a one-dimensional time domain signal in which the power (variance) of one class is maximized while the power of the other class is minimized. Unlike PCA, CSP handles two classes at the same time and simultaneously diagonalizes the covariance matrices of both classes [9]. Moreover, the CSP algorithm was proven to be efficient in BCI competitions [10, 11].

Although CSP is a powerful and simple technique, it has some drawbacks. CSP optimizes the average power ratio of the two classes, and therefore, it requires only one average covariance matrix for each class. This may be a problem when addressing non-stationary signals such as EEG signals because the covariance matrix of an EEG signal may change over time due to artifacts such as changes in EEG electrode-skin impedances, muscular activities or user background EEG activity [7]. Representing all of the epochs of a class in a training set by only one average covariance matrix should result in inaccurate spatial filters.

Another disadvantage of CSP is its strict fitness function. CSP does not allow different types of fitness functions, which may be more useful in different situations [12]. CSP attempts to optimize the Rayleigh quotient, i.e., the ratio of average variances of the two classes, which is very sensitive to outliers that cause over fitting [13].

There are numerous methods for increasing the robustness of CSP. Recently, a method called regularized CSP (RCSP) has been proposed, which aims to compute more robust spatial patterns by adding a regularization term to the CSP formula [5, 7, 14]. The RCSP method uses some a priori knowledge and imposes various constraints in the CSP’s formulation to obtain more robust spatial filters [12]. For example, Lotte [14] proposed spatially regularized CSP (SRCSP). He used the a priori knowledge that neighboring neurons tend to have similar functions, which supports the hypothesis that neighboring electrodes should measure similar brain signals. Another example of regularizing CSP is stationary CSP (sCSP), which was proposed by Samek et al. [7]. sCSP assumes that non-stationeries in EEG data come from processes that are not task related, such as eye movements or electrode artifacts. Another study on robust CSP is CSP-L1 by Wang et al. [8], who attempted to express the CSP formulation in L1 norm. He states that the original formulation of CSP was L2-norm, which implies that CSP was sensitive to outliers. Wang attempted to optimize the proposed alternative CSP formulation using an iterative algorithm.

In this study, we propose a general framework called spatial filter network (SFN), which calculates optimal spatial filters using a neural network approach. With SFN, each epoch in a training set is given to the network for learning the optimal spatial filters, unlike CSP, which only uses one average covariance matrix for each class. Additionally, SFN is trained to directly increase the classification accuracy, whereas the purpose of the CSP method is to maximize the given optimization function, which indirectly increases the classification accuracy. In this paper, the CSP algorithm is described first. Then, SFN is introduced along with its network structure and training methods. Finally, both methods are applied to motor imagery data and the results are compared.

The remainder of this paper is organized as follows. In the Materials and Methods section, the standard CSP method and its multi-class version are briefly reviewed. Then, the proposed SFN method and the proposed training methods are described with a simple toy data example. Finally, the motor imagery data set used in this study and the EEG preprocessing routine are described. The Results section summarizes the evaluations and the results of the study. The Discussion section investigates the advantages/disadvantages of the SFN. Finally, the Conclusion section summarizes the study and presents directions for future work.

## Materials and Methods

### Common spatial patterns

CSP is a widely used technique for obtaining good spatial resolution and discrimination between different classes of motor imagery signals. In general, a motor imagery experiment consists of epochs, in which the user imagines one type of motor imagery task requested on the screen. An epoch can be one of two classes: *C*_1_ and *C*_2_. (i.e., left hand—right hand). Let *X*_*C*,*i*_ ∈ ℝ^*NxT*^ represent an epoch, where *C* is the class of the epoch, *i* is the epoch number belonging to class *C*, *N* is the number of EEG channels, and *T* is the number of samples in the epoch. Note that *X*_*C*,*i*_ should be a zero average signal (i.e., band pass filtered). Let $\vec{w}\in {\mathbb{R}}^{Nx1}$ be a vector in *N*-dimensional space. A projection of an epoch onto this vector will be

$$\begin{array}{c} {\vec{y}}_{C,i}={\vec{w}}^{T}X_{C,i} \end{array}$$ (1)

where ${\vec{y}}_{C,i}\in {\mathbb{R}}^{1xT}$ denotes the projection of epoch *X*_*C*,*i*_ and *T* is the transpose operation. The projected signal power *P*_*C*,*i*_ can be written as follows:

$$\begin{array}{c} P_{C,i}={\vec{y}}_{C,i}{\vec{y}}_{C,i}^{T}={\vec{w}}^{T}X_{C,i}X_{C,i}^{T}\vec{w} \end{array}$$ (2)

Let *R*_*C*,*i*_ ∈ ℝ^*NxN*^ be the covariance matrix of the band pass-filtered signal *X*_*C*,*i*_ and ${\bar{R}}_{C}\in {\mathbb{R}}^{NxN}$ be the average covariance matrix of class *C*:

$$\begin{array}{c} R_{C,i}=\frac{X_{C,i}X_{C,i}^{T}}{tr(X_{C,i}X_{C,i}^{T})}\;{\bar{R}}_{C}=\frac{1}{n_{C}}\sum_{i\in C}^{n_{C}}R_{C,i} \end{array}$$ (3)

where *tr* is the trace function and *n*_*C*_ is the number of epochs in *C*. Let the average power of class *C* be ${\bar{P}}_{C}$. Then, ${\bar{P}}_{C}$ is calculated as follows:

$$\begin{array}{c} {\bar{P}}_{C}=\frac{1}{n_{C}}\sum_{i\in C}^{n_{C}}{\vec{w}}^{T}X_{C,i}X_{C,i}^{T}\vec{w}=\frac{1}{n_{C}}\sum_{i\in C}^{n_{C}}{\vec{w}}^{T}R_{C,i}\vec{w}={\vec{w}}^{T}{\bar{R}}_{C}\vec{w} \end{array}$$ (4)

For the two classes (*C* = 1,2) case, CSP searches for the maximum power ratio of the classes on the projected *w* axis. Thus, the average power of one class is maximized while that of the other class is minimized. In other words, the spatial filter should maximize the following Rayleigh quotient problem [7]:

$$\begin{array}{c} \underset{\vec{w}}{arg max}\frac{{\vec{w}}^{T}{\bar{R}}_{1}\vec{w}}{{\vec{w}}^{T}{\bar{R}}_{2}\vec{w}} \end{array}$$ (5)

For any $\vec{w}$ that maximizes Eq (5), the denominator can be set to a constant value *c* by a scalar coefficient without changing the ratio. Thus, maximization of the Rayleigh quotient can be retranslated into a constrained optimization problem:

$$\begin{array}{c} maximize\;{\vec{w}}^{T}{\bar{R}}_{1}\vec{w},\;subject\;to\;\;\;\;{\vec{w}}^{T}{\bar{R}}_{2}\vec{w}=c \end{array}$$ (6)

The above constrained optimization problem can be solved using the Lagrange multiplier method [15]:

$$\begin{array}{c} L\left(\lambda ,\vec{w}\right)={\vec{w}}^{T}{\bar{R}}_{1}\vec{w}-\lambda \left({\vec{w}}^{T}{\bar{R}}_{2}\vec{w}-c\right) \end{array}$$ (7)

$$\begin{array}{c} \frac{\partial L(\lambda ,\vec{w})}{\partial \vec{w}}=2{\vec{w}}^{T}{\bar{R}}_{1}-\lambda \left(2{\vec{w}}^{T}{\bar{R}}_{2}\right)=0 \end{array}$$ (8)

where *λ* is the Lagrange multiplier. Because ${\bar{R}}_{C}$ is a symmetric matrix, the above equation can be written as a standard eigenvalue problem:

$$\begin{array}{c} \left({{\bar{R}}_{2}}^{-1}{\bar{R}}_{1}\right)\vec{w}=\lambda \vec{w} \end{array}$$ (9)

According to Eq (9), *w*, which maximizes the Rayleigh quotient, is the eigenvector that corresponds to the largest eigenvalue of $\left({{\bar{R}}_{2}}^{-1}{\bar{R}}_{1}\right)$.

The CSP spatial filter *W*_*CSP*_ ∈ ℝ^*MxN*^ matrix is constructed by taking *M* = 2*m* (*M* ≤ *N*) eigenvectors corresponding to the *m* largest and *m* smallest eigenvalues:

$$\begin{array}{c} W_{CSP}={\left[{\vec{w}}_{\lambda_{1}},...{\vec{w}}_{\lambda_{m}}......{\vec{w}}_{\lambda_{N-m+1}},...{\vec{w}}_{\lambda_{N}}\right]}^{T} \end{array}$$ (10)

where *w*_*λ*_*i*__ is the eigenvector that corresponds to the eigenvalue *λ*_*i*_. Any epoch *X*_*C*,*i*_ is spatially filtered by

$$\begin{array}{c} Z_{C,i}=W_{CSP}X_{C,i} \end{array}$$ (11)

where *Z*_*C*,*i*_ ∈ ℝ^*MxT*^ is the spatially filtered signal. Band power (variance) is used as a feature for the classifier. For an epoch *i*, the CSP feature vector is given by

$$\begin{array}{c} f\vec{cs}p_{C,i}^{k}=log\left(\frac{var\left(Z_{C,i}^{k}\right)}{\sum_{l=1}^{2m}var\left(Z_{C,i}^{l}\right)}\right)k=1,2,\ldots M \end{array}$$ (12)

where $f\vec{cs}p_{C,i}^{k}$ is the *k* ^*th*^ feature of feature vector $f\vec{cs}p_{C,i}\in {\mathbb{R}}^{Mx1}$ that belongs to epoch *i* and $Z_{C,i}^{k}$ is the *k* ^*th*^ row of *Z*_*C*,*i*_. Here, the logarithm of the variance ratio is calculated to approximate the distribution of the features to a normal distribution [9]. Next, the features are used to train a linear classifier.

### Extending CSP to multiclass

The optimization function of CSP is defined for two classes. When there are more than two motor imagery classes (e.g., *left hand*, *right hand*, *foot*, *tongue*, etc..), the CSP method requires some modifications. Extending CSP to multiclass is achieved via a combination of classes, either converting a multiclass problem to several binary problems or computing CSP for one class versus all other classes, called *One Versus the Rest (OVR) CSP* [16]. The OVR-CSP method aims to maximize the power of one class versus the total power of the rest of the classes. The OVR-CSP for class *c* is calculated as follows:

$$\begin{array}{c} {\vec{w}}_{c}=\underset{\vec{w}}{\text{arg}\;\text{max}}\frac{{\vec{w}}^{T}{\bar{R}}_{c}\vec{w}}{\sum_{j\neq c}^{C}{\vec{w}}^{T}{\bar{R}}_{j}\vec{w}} \end{array}$$ (13)

where *C* is the total number of classes and ${\vec{w}}_{c}$ is the vector that maximizes the Rayleigh ratio between class *c* and the other classes. The CSP matrix is constructed by calculating the above equation for all classes. Note that OVR-CSP is a generalization for the CSP method, which is equal to (Eq 5) for the two classes (*C* = 2) case.

### Spatial filter network (SFN)

**Fig 1:**
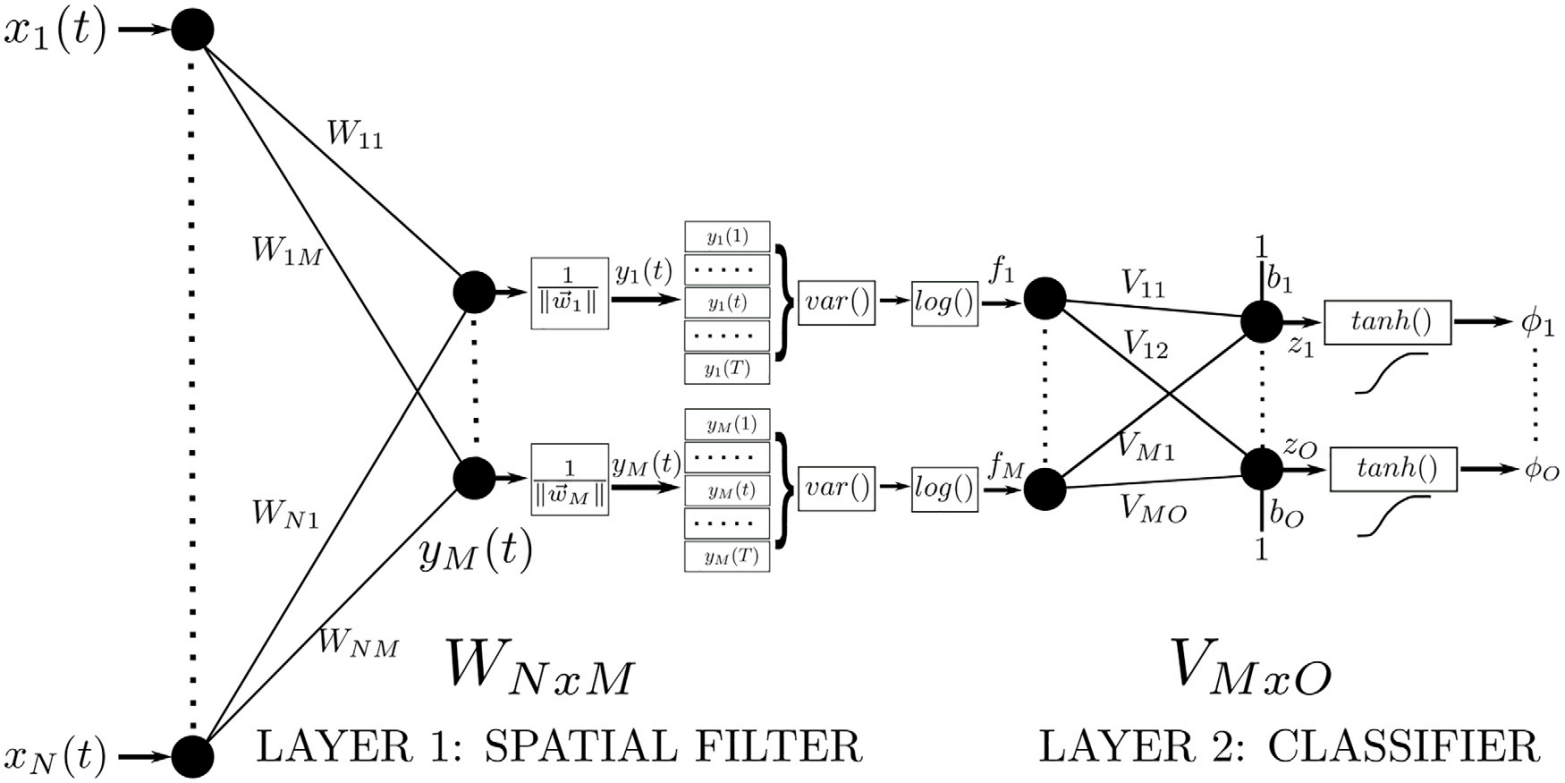
Structure of the proposed spatial filter network (SFN).

**Fig 2:**
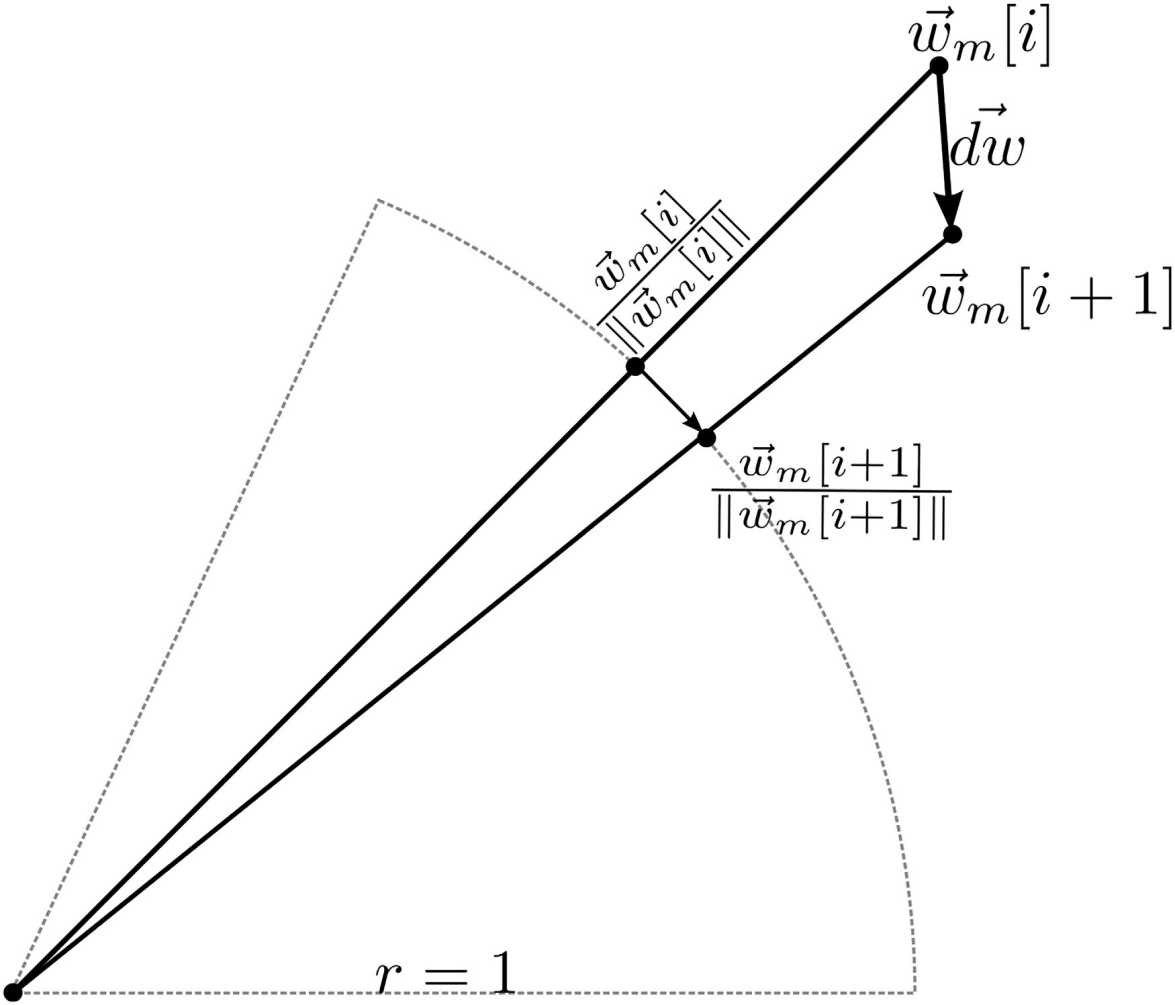
A representative figure of searching the optimal spatial filter over the hypersphere of unit radius.

In this section, the proposed spatial filter network (SFN) model is introduced. SFN is depicted in Fig 1. SFN consists of a spatial filter layer (Layer-1) and a classification layer (Layer-2), which are connected to each other with non-linear mapping functions. Layer-1 is formed with a spatial filtering matrix *W* and feature extraction functions. The input-output relations of the spatial filter layer are given below:

$$\begin{array}{c} y_{m}\left(t\right)=\frac{\sum_{n=1}^{N}W_{nm}\vec{x}\left(t\right)}{\sqrt{\sum_{n=1}^{N}W_{nm}^{2}}}=\frac{{\vec{w}}_{m}^{T}}{∥{\vec{w}}_{m}∥}\vec{x}\left(t\right) \end{array}$$ (14)

where $\vec{x}\left(t\right)\in {\mathbb{R}}^{Nx1}$ is the input data at time *t* of the EEG epoch with *N* channels and *T* samples, 0<t≤T. ${\vec{w}}_{m}\in {\mathbb{R}}^{Nx1}$ is the *m* ^*th*^ column of the spatial filter matrix *W* ∈ ℝ^*NxM*^, in which each column is a spatial filter and *y*_*m*_(*t*) is the output data at time *t* for the *m* ^*th*^ spatial filtering output. ${\vec{w}}_{m}$ is divided by its norm to ensure that the input signal is spatially filtered with unit norm filters. Thanks to the $1/\|{\vec{w}}_{m}\|$ block, SFN searches for optimal spatial filters over the surface a hypersphere in N dimensions. As shown in Fig 2, the training algorithm, which moves ${\vec{w}}_{m}$ by *d*_*w*_, actually moves the spatial filter over the hypersphere.

Spatial filtering is an important step in motor imagery classification. Redundant data that belong to irrelevant channels are weakened with spatial filtering. Spatial filtering is applied to the input data, and outputs ${\vec{y}}_{m}\left(t\right)$ are stored until all samples in one epoch are counted. After the spatial filtering phase, Layer-1 calculates the feature vector to be an input for Layer-2.

$$\begin{array}{c} \vec{f_{m}}=log\left(var\left({\vec{y}}_{m}\right)\right)=log\left(\frac{1}{T}\sum_{t=1}^{T}{\left(y_{m}\left(t\right)-\mu_{{\vec{y}}_{m}}\right)}^{2}\right)\;m=1,2,...M \end{array}$$ (15)

where *var*() is the variance operation and *log*() is the natural logarithm function. Because input data *X* are zero mean, $\mu_{{\vec{y}}_{m}}$ will be zero. $\vec{f}\in {\mathbb{R}}^{Mx1}$ is the feature vector for a given epoch. Note that the logarithm is used as in CSP to approximate the distribution of the features to a normal distribution [9].

Layer-2 of SFN is the classification layer. An *M*-dimensional input feature vector (*f*) is mapped to an *O*-dimensional output vector *z*. The input-output relation of Layer-2 is given below:

$$\begin{array}{c} \vec{z}=V^{T}\vec{f}+\vec{b} \end{array}$$ (16)

$$\begin{array}{c} \vec{\Phi }=tanh\left(\vec{z}\right) \end{array}$$ (17)

where *V* ∈ ℝ^*MxO*^ is the weight matrix, $\vec{b}\in {\mathbb{R}}^{Ox1}$ is the bias vector, *tanh*() is the tangent hyperbolic function used for clamping $\vec{z}$ to the range (−1, + 1), and $\vec{\Phi }$ is the network output. Labeling of an input epoch *X* ^*k*^ differs for binary and multi-category cases. For the two classes (binary) case, a single output neuron (*O* = 1) with a threshold value is used. Because *tanh* is used as an activation function, the threshold value will be 0. For a multi-classes case, the number of output neurons should be equal to the number of classes (*O* = *C*). In this case, the class label of an input epoch *X* ^*k*^ is assigned by selecting the output neuron with the highest $\vec{\Phi }$ value among all output neurons.

$$\begin{array}{c} Class\left(X^{k}\right)=\underset{c}{arg max}\left(\vec{\Phi_{c}^{k}}\right)\;c=1,2,...C \end{array}$$ (18)

Because the class label is generated by selecting the maximum output value, the resulting classifier is called a *linear machine*, in which no ambiguous region exists. A linear machine divides the feature space into *C* decision regions, with $\vec{\phi_{c}}$ being the largest discriminant if *X* ^*k*^ in region *R*_*c*_ [17].

### Training of SFN

We used two methods to train the SFN: Backpropagation [18] and Levenberg-Marquardt. Both methods use partial derivatives to optimize the spatial filter coefficients *W*_*ij*_. For the Backpropagation method, the coefficients are updated after each epoch is presented to SFN, whereas for the Levenberg-Marquardt method, updating is performed after all epochs in the training set are presented to the network. For both methods, the error function is calculated according to the network output and the class label of the epoch presented to the network. Additionally, the initial weights for *W*, *V* and $\vec{b}$ are set randomly with a normal distribution *σ* = 0.1 and *μ* = 0.

#### Error function

SFN requires an error function *E* to optimize the spatial filter *W*_*nm*_ and classifier coefficients *V*_*mo*_. *E* should be minimum when SFN successfully discriminates different classes in the training set. We used the Euclidean distance between the target class vector $\vec{T_{c}}$ and SFN output vector $\vec{z}$ as the error measure. The error value of the *k* ^*th*^ epoch is given by:

$$\begin{array}{c} E^{k}=\frac{1}{2}\sum_{o=1}^{O}{\left(e_{o}^{k}\right)}^{2}=\frac{1}{2}\sum_{o=1}^{O}{\left({\vec{\Phi }}_{o}^{k}-{\vec{D}}_{o}^{k}\right)}^{2}\;,1\leq k\leq K \end{array}$$ (19)

where *k* is the current epoch number presented to the SFN, ${\vec{\phi }}^{k_{o}}$ is the *o* ^*th*^ output of the SFN, ${\vec{D}}^{k_{o}}$ is the *o* ^*th*^ element of the target vector when the *k* ^*th*^ epoch is given to the network, and *K* is the total number of epochs in the training set. SFN is trained iteratively such that the total error of the network for the training class should be minimized.

#### Backpropagation method

The Backpropagation (BP) method adapts all weights of a neural network to minimize the error on a set of vectors belonging to a pattern recognition problem [19]. The BP learning rule is based on gradient descent. The weights are initialized with random values and changed in a direction to reduce the error [17]. In this study, the BP method is used to optimize both the spatial filter layer and the classifier layer. In a classical pattern recognition problem, a feature vector is given to the feed-forward neural network and the weights of the network are updated according to the feature’s class and the network’s output. However, the proposed training method accepts all of the samples in an epoch and updates the weights when an epoch is completely given to the network. For each layer, the learning rule of SFN is given by

$$\begin{array}{c} \begin{array}{c} W_{nm}=W_{nm}-\mu \frac{\partial E}{\partial W_{nm}}\\ V_{mo}=V_{mo}-\mu \frac{\partial E}{\partial V_{mo}}\\ {\vec{b}}_{o}={\vec{b}}_{o}-\mu \frac{\partial E}{\partial {\vec{b}}_{o}} \end{array} \end{array}$$ (20)

where *V*_*mo*_ and *W*_*nm*_ are the weights of the classifier and spatial filter layers, respectively, *b*_*o*_ is the bias value for the second layer of the network, and *μ* is the learning rate parameter. The partial derivatives for each layer are calculated using the chain rule:

$$\begin{array}{c} \frac{\partial E}{\partial V_{m,o}}=\frac{\partial E}{\partial {\vec{\Phi }}_{o}}\frac{\partial {\vec{\Phi }}_{o}}{\partial {\vec{z}}_{o}}\frac{\partial {\vec{z}}_{o}}{\partial V_{m,o}} \end{array}$$ (21)

$$\begin{array}{c} \frac{\partial E}{\partial {\vec{b}}_{o}}=\frac{\partial E}{\partial {\vec{\Phi }}_{o}}\frac{\partial {\vec{\Phi }}_{o}}{\partial {\vec{z}}_{o}}\frac{\partial {\vec{z}}_{o}}{\partial {\vec{b}}_{o}} \end{array}$$ (22)

$$\begin{array}{c} \frac{\partial E}{\partial W_{n,m}}=\sum_{o=1}^{O}\frac{\partial E}{\partial {\vec{\Phi }}_{o}}\frac{\partial {\vec{\Phi }}_{o}}{\partial {\vec{z}}_{o}}\frac{\partial {\vec{z}}_{o}}{\partial {\vec{f}}_{m}}\sum_{t=1}^{T}\frac{\partial {\vec{f}}_{m}}{\partial {\vec{y}}_{m}\left(t\right)}\frac{\partial {\vec{y}}_{m}\left(t\right)}{\partial W_{n,m}} \end{array}$$ (23)

Note that the error of any output propagates to all weights in the spatial filter layer. Because the classifier layer output and error value are calculated after all of the samples in one epoch are counted, backpropagation should be calculated after the epoch is fully presented to the SFN. The required backward equations (derivatives) are given in Eqs (24)–(28):

$$\begin{array}{c} \frac{\partial E}{\partial {\vec{\Phi }}_{o}}={\vec{\Phi }}_{o}-\vec{D} \end{array}$$ (24)

$$\begin{array}{c} \frac{\partial {\vec{\Phi }}_{o}}{\partial {\vec{z}}_{o}}=1-{\left({\vec{\Phi }}_{o}\right)}^{2} \end{array}$$ (25)

$$\begin{array}{c} \frac{\partial {\vec{z}}_{o}}{\partial V_{m,o}}={\vec{f}}_{m}\;\frac{\partial {\vec{z}}_{o}}{\partial {\vec{f}}_{m}}=V_{m,o}\;\frac{\partial {\vec{z}}_{o}}{\partial {\vec{b}}_{o}}=1 \end{array}$$ (26)

$$\begin{array}{c} \frac{\partial {\vec{f}}_{m}}{\partial {\vec{y}}_{m}\left(t\right)}=\frac{1}{\frac{1}{T}\sum_{t=1}^{T}{\left({\vec{y}}_{m}\left(t\right)\right)}^{2}}\frac{2}{T}{\vec{y}}_{m}\left(t\right) \end{array}$$ (27)

$$\begin{array}{c} \frac{\partial {\vec{y}}_{m}\left(t\right)}{\partial W_{nm}}=\frac{\|{\vec{w}}_{m}\|{\vec{x}}_{n}\left(t\right)-W_{nm}{\vec{y}}_{m}\left(t\right)}{\|{\vec{w}}_{m}{\|}^{2}} \end{array}$$ (28)

With the BP method, the weights (*W*, *V* and $\vec{b}$) are updated after each epoch. Training SFN with backpropagation is listed in Algorithm 1. Here, *itr* refers to the iteration number, *MAXITR* is the maximum number of iterations, *EMIN* is the minimum error value to continue iterations, and *W* ^0^, *V* ^0^ and ${\vec{b}}^{0}$ are the initial values for *W*, *V* and $\vec{b}$, respectively.

**Algorithm 1 Pseudo-code for BP algorithm**

*itr* ← 0, *W* ← *W* ^0^, *V* ← *V* ^0^, $\vec{b}\leftarrow {\vec{b}}^{0}$

**while** *itr* + + < *MAXITR* **and** *E* > *EMIN* **do**

  Select an epoch from training set

  Calculate network output (Eqs 14–17)

  Calculate error function *E* (Eq 19)

  Calculate ∂*E*/∂*V*_*mo*_,$\partial E/\partial {\vec{b}}_{o}$ and ∂*E*/∂*W*_*nm*_ (Eqs 21–23)

  Update network weights (Eq 20)

**end while**

#### Levenberg–Marquardt method

The Levenberg–Marquardt (LM) algorithm [20, 21] iteratively generates a solution for minimizing a non-linear problem. It is fast and has stable convergence [22]. Unlike the steepest descent method that the back-propagation algorithm uses, the LM method is an approximation to Newtons Method [23]. Let $E(\vec{q})$ be the total error that is iteratively minimized by the proposed method:

$$\begin{array}{c} E\left(\vec{q}\right)=\frac{1}{2}\sum_{k=1}^{K}\sum_{o=1}^{O}{\left(e_{o}^{k}\right)}^{2} \end{array}$$ (29)

where $e_{o}^{k}$ is the error at the output *o* for the epoch number *k* and $\vec{q}\in {\mathbb{R}}^{NM+O(M+1)}$ is the vector of all weights forming the SFN:

$$\begin{array}{c} e_{o}^{k}=D_{o}^{k}-\Phi_{o}^{k} \end{array}$$ (30)

$$\begin{array}{c} \vec{q}=\left[W_{11},W_{12}...W_{NM},V_{11},V_{12},...,V_{MO},b_{1},b_{2},....b_{O}\right] \end{array}$$ (31)

The Levenberg–Marquardt method aims to minimize $E(\vec{q})$ according to the following update rule:

$$\begin{array}{c} {\vec{q}}_{i+1}={\vec{q}}_{i}-{\left(J_{i}^{T}J_{i}+\mu I\right)}^{-1}J_{i}{\vec{e}}_{i} \end{array}$$ (32)

where *i* is the iteration number, $\vec{e}\in {\mathbb{R}}^{KO}$ is a vector that holds the errors of all outputs, *J* ∈ ℝ^(*NM*+*O*(*M*+1))*x*(*KO*)^ is the Jacobian matrix, and *μ* is called the combination coefficient. When *μ* is very small, the algorithm works as the Gauss-Newton method, but when *μ* is large, the algorithm turns to the steepest descend [23] method. $\vec{e}$ and *J* are introduced below:

$$\begin{array}{c} e={\left[e_{1}^{1},e_{2}^{1},...,e_{O}^{1}...,e_{1}^{K},e_{2}^{K},...,e_{O}^{K}\right]}^{T} \end{array}$$ (33)

$$\begin{array}{c} J=(\begin{array}{ccccc} \frac{\partial e_{1}^{1}}{\partial {\vec{q}}_{1}} & \frac{\partial e_{1}^{1}}{\partial {\vec{q}}_{2}} & ... & \frac{\partial e_{1}^{1}}{\partial {\vec{q}}_{NM+O(M+1)}} & \\ \frac{\partial e_{2}^{1}}{\partial {\vec{q}}_{1}} & \frac{\partial e_{2}^{1}}{\partial {\vec{q}}_{2}} & ... & \frac{\partial e_{2}^{1}}{\partial {\vec{q}}_{NM+O(M+1)}} & \\ ... & ... & ... & ...\\ \frac{\partial e_{O}^{1}}{\partial {\vec{q}}_{1}} & \frac{\partial e_{O}^{1}}{\partial {\vec{q}}_{2}} & ... & \frac{\partial e_{O}^{1}}{\partial {\vec{q}}_{NM+O(M+1)}} & \\ ... & ... & ... & ...\\ \frac{\partial e_{1}^{K}}{\partial {\vec{q}}_{1}} & \frac{\partial e_{1}^{K}}{\partial {\vec{q}}_{2}} & ... & \frac{\partial e_{1}^{K}}{\partial {\vec{q}}_{NM+O(M+1)}} & \\ \frac{\partial e_{2}^{K}}{\partial {\vec{q}}_{1}} & \frac{\partial e_{2}^{K}}{\partial {\vec{q}}_{2}} & ... & \frac{\partial e_{2}^{K}}{\partial {\vec{q}}_{NM+O(M+1)}} & \\ ... & ... & ... & ...\\ \frac{\partial e_{O}^{K}}{\partial {\vec{q}}_{1}} & \frac{\partial e_{O}^{K}}{\partial {\vec{q}}_{2}} & ... & \frac{\partial e_{O}^{K}}{\partial {\vec{q}}_{NM+O(M+1)}} & \end{array}) \end{array}$$ (34)

When calculating the Jacobian matrix, the chain rule is applied to $\partial e/\partial \vec{q}$, and the following equations are obtained:

$$\begin{array}{c} \frac{\partial e_{o}}{\partial V_{mo}}=\frac{\partial e_{o}}{\partial {\vec{\Phi }}_{o}}\frac{\partial {\vec{\Phi }}_{o}}{\partial {\vec{z}}_{o}}\frac{\partial {\vec{z}}_{o}}{\partial V_{mo}} \end{array}$$ (35)

$$\begin{array}{c} \frac{\partial e_{o}}{\partial {\vec{b}}_{o}}=\frac{\partial e_{o}}{\partial {\vec{\Phi }}_{o}}\frac{\partial {\vec{\Phi }}_{o}}{\partial {\vec{z}}_{o}}\frac{\partial {\vec{z}}_{o}}{\partial {\vec{b}}_{o}} \end{array}$$ (36)

$$\begin{array}{c} \frac{\partial e_{o}}{\partial W_{nm}}=\frac{\partial e_{o}}{\partial {\vec{\Phi }}_{o}}\frac{\partial {\vec{\Phi }}_{o}}{\partial {\vec{z}}_{o}}\frac{\partial {\vec{z}}_{o}}{\partial {\vec{f}}_{m}}\sum_{t=1}^{T}\frac{\partial {\vec{f}}_{m}}{\partial {\vec{y}}_{m}\left(t\right)}\frac{\partial {\vec{y}}_{m}\left(t\right)}{\partial W_{nm}} \end{array}$$ (37)

Note that from (Eq 30), ∂*e*_*o*_/∂*ϕ*_*o*_ equals -1. Other terms may be calculated using Eqs (24)–(28). At the end of each epoch, the network outputs and error values are calculated, and then the Jacobian matrix is constructed. The LM method updates the network weights after all of the epochs in the training set are presented to the SFN. *μ* is increased or decreased with each iteration such that the convergence rate is adjusted. Additionally, if the total error of the network increases with new weights after updating, the LM algorithm sets the weights to their previous values and slows the convergence rate. Training of SFN with the LM method is demonstrated using the Algorithm 2. Here, *μ*_0_ is the initial value for the combination coefficient *μ* and *β* is the multiplier (divider) of *μ* for increasing (decreasing) it. For other terms, please refer to the description of the BP Algorithm 1. Note that, depending on the convergence of the network, the algorithm slightly changes to the steepest-descend or Gauss-Newton method by adjusting the value of *μ*.

**Algorithm 2 Pseudo-code for LM algorithm**

*itr* ← 0, *μ* ← *μ*_0_, *W* ← *W* ^0^, *V* ← *V* ^0^, $\vec{b}\leftarrow {\vec{b}}^{0}$

**while** *itr* + + < *MAXITR* **and** *E* > *EMIN* **do**

**for all** epoch in Training Set **do**

   Calculate network output (Eqs 14–17)

   Calculate error for all outputs $e_{o}^{k}$ (Eq 30)

   Calculate Jacobian matrix rows for the current epoch (Eq 34)

**end for**

  Calculate total error (Eq 29)

**if** *E*_*itr*_ < *E*_*itr*−1_ **then**

*μ* ← *μ*/*β*

**else**

*μ* ← *μ* * *β*

   Revert to previous weights *q*_*itr*_ ← *q*_*itr*−1_

**end if**

  Calculate new weights vector *q*_*itr*_ (Eq 32)

  Update network weights *W*, *V* and $\vec{b}$ (Eq 31)

**end while**

### Running SFN with toy data

**Fig 3:**
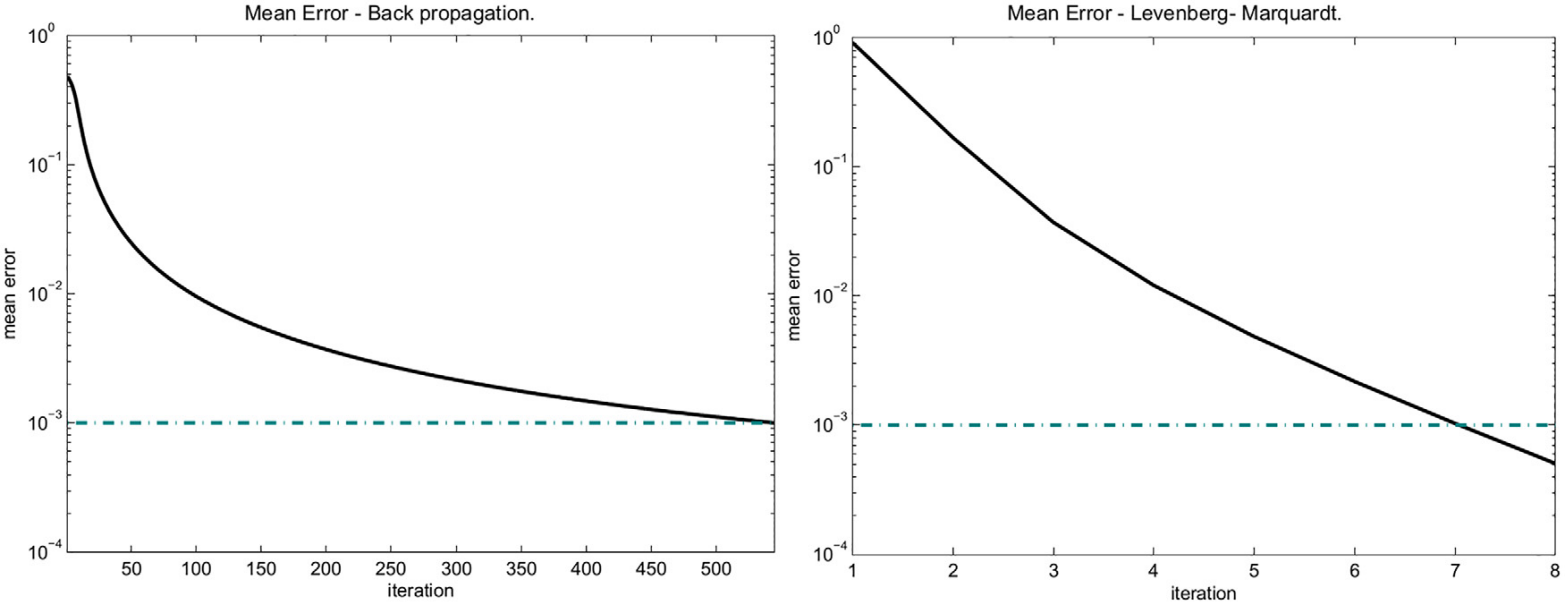
Convergence of Backpropagation and Levenberg–Marquardt algorithms to the desired error (*EMIN*, dashed line) for 2 classes toy data example.

In this section, we test the SFN using generated toy data for 2 and 4 classes. Each class in the generated data has a different covariance matrix. For visualization purposes, the dimensions of the toy data and the size of the output neuron of the spatial filter layer were set to 2 (*N* = 2, *M* = 2). Note that the class label of an epoch in the toy data was randomly selected. Therefore, the number of epochs for each class will be approximately identical. Additionally, epoch data were generated with zero mean, fixed covariance matrices specific to each class using the Matlab command *mvnrnd* [24]. For both data sets, the epoch dimension (*NxT*) was set to 2*x*100 and the total number of epochs (*K*) was set to 100. SFN was trained with the BP and LM algorithms. The network parameters are as follows: *μ* = 10^−3^ for the BP method and *μ*_0_ = 100, *β* = 2 for the LM method. Both methods successfully converged to the desired error value (*EMIN* = 10^−3^). As expected, the LM algorithm converged faster than the BP algorithm. The convergence of the two methods for the 2-classes case is shown in Fig 3.

**Fig 4:**
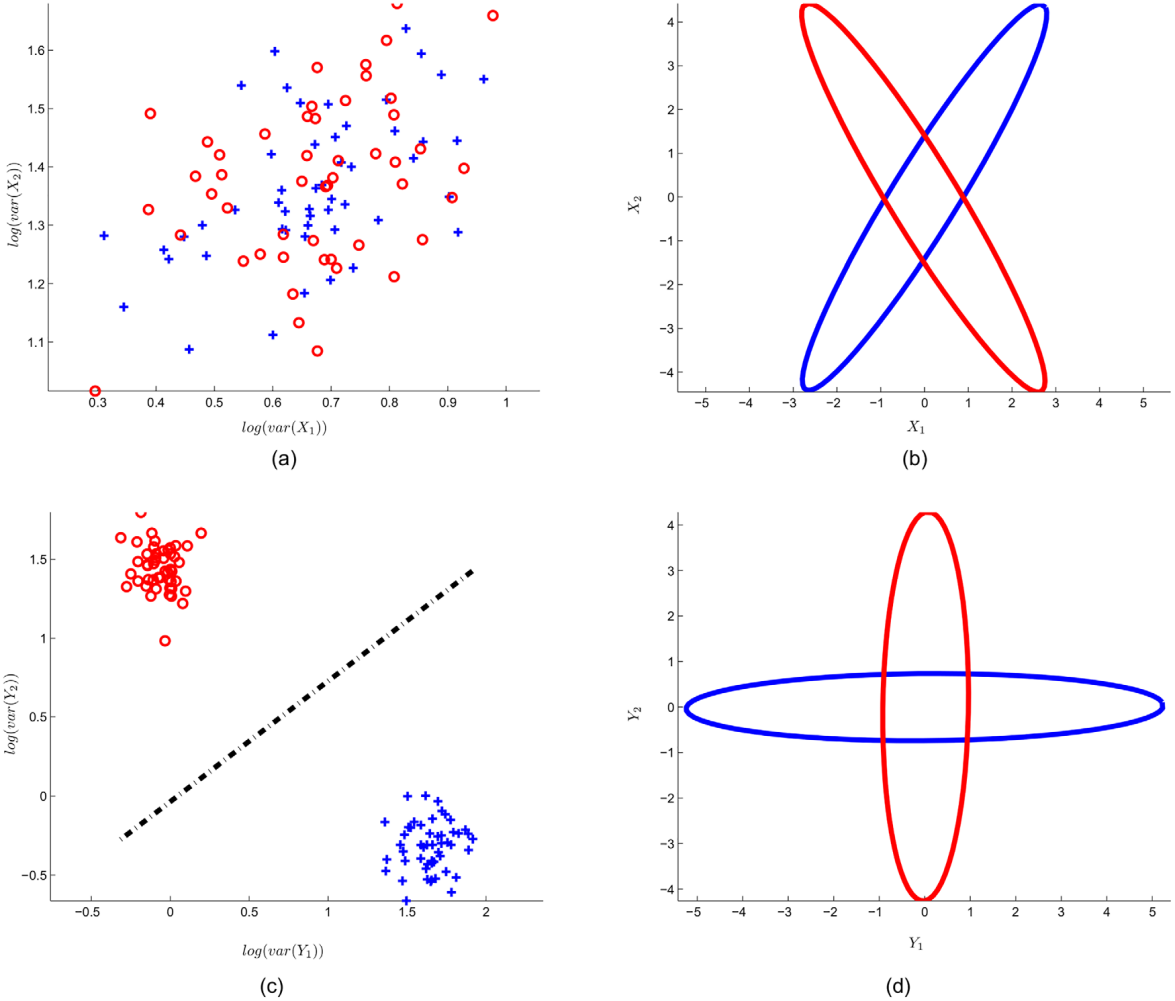
Input data and SFN output data for toy data with 2 classes. (a) log-variance feature for 2-dimensional input data. Note that each point represents an epoch that belongs to class 1 (red circle) or class 2 (blue plus). (b) Enclosing ellipses represent the input data. (c) SFN spatial filter layer output (*f*) with generated class border (black dashed line) of the classifier layer. (d) Enclosing ellipses represent the spatially filtered input data (*y*).

Fig 4 presents the input data and the SFN output data for the 2-classes case. In this Figure, (a) illustrates the log-variance feature of the input data:

$$\begin{array}{c} \begin{array}{c} f_{1}\left(X^{k}\right)=log\left(var\left(\left[X_{11}^{k},X_{12}^{k},....X_{1T}^{k}\right]\right)\right)\\ f_{2}\left(X^{k}\right)=log\left(var\left(\left[X_{21}^{k},X_{22}^{k},....X_{2T}^{k}\right]\right)\right) \end{array} \end{array}$$ (38)

where *X* ^*k*^ is the *k* ^*th*^ epoch. Each red (circle) and blue (plus) point represent the epoch with class 1 or 2, respectively. In (b), the input data calculated by principle components are enclosed with ellipses. Note that the input data have the same variance in each dimension. (c) Shows the effect of the SFN spatial filter layer, i.e., scattering of spatially filtered data ($f_{m}^{k}$). The spatial filter layer successfully separates the two classes. Here, the black dashed line shows the between-class border created by SFN classifier layer. In (d), the effect of SFN spatial filtering is shown with enclosing ellipses. Note that spatial filtering manipulates the data, in which the features that belong to any class have maximum variance in one dimension, and they have minimum variance in the other dimension.

**Fig 5:**
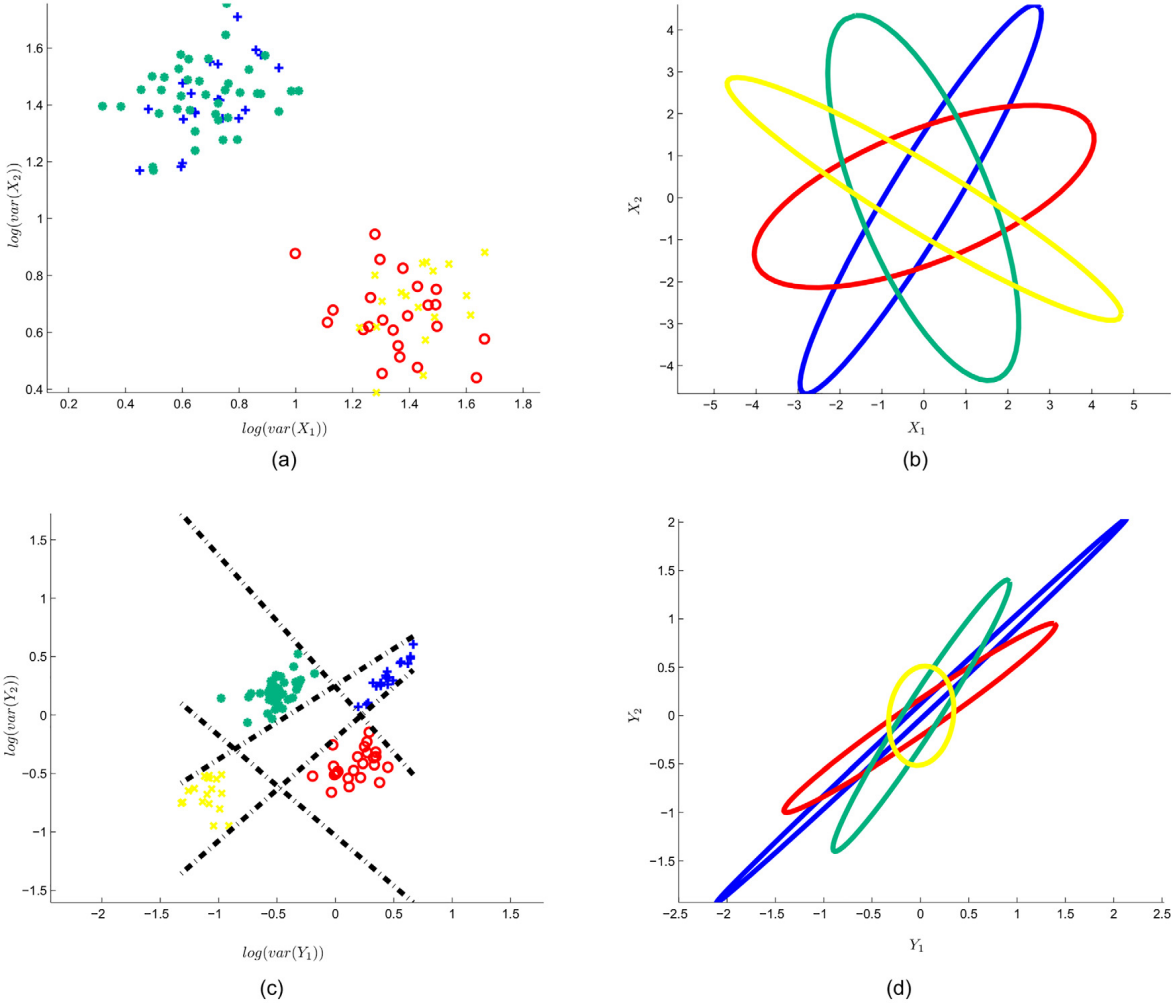
Input data and SFN output data for toy data with 4-classes. (a) log-variance feature for 2-dimensional input data. Note that each point represents an epoch that belongs to class 1 (red circle), class 2 (blue plus), class 3 (green asterisk) or class 4 (yellow cross). (b) Enclosing ellipses represent the input data. (c) SFN spatial filter layer output (*f*) with generated class borders (black dashed lines) of the classifier layer. (d) Enclosing ellipses represent the spatially filtered input data (*y*).

The SFN output for the 4-classes toy data is shown in Fig 5. In (a), the log-variance features of the 4 classes are shown. (b) Illustrates the enclosing ellipses for each class. The spatial filter output is given in (c). As for the 2-classes case, the spatial filter layer of SFN has two outputs (*M* = 2) such that we are able to visualize the output data. However, a larger output dimension may be used for data with more complex scattering. Because the SFN classifier has 4 outputs (*O* = 4), there are 4 class borders, which are drawn in (c). Note that, as a result of the selected target vectors in the one-versus-rest style, each borderline separates one class from the other classes. As shown in (d), the spatial filter layer manipulated the input data such that each class will have a different variance vector.

### EEG data sets

In this study, we used two publicly available EEG motor imagery data sets: BCI competition III Data Set IVa and BCI competition III Data Set IIIa [11]. Data Set IVa consists of EEG recordings of 5 subjects who performed motor imagery of the right hand and foot. A total of 118 electrodes were used for recording EEGs with a sample rate of 100 Hz. There are 280 trials for each subject. However, the numbers of training and test sets differ for each subject: 168, 224, 84, 56 and 28 trials are the sizes of the training sets for subjects labeled as *aa*, *al*, *av*, *aw* and *ay*, respectively, and the remaining trials form the test set. Note that no validation set was used for network training, and we applied the test set after SFN approached the target error value with the training set in the training phase. However, we limited the iteration number and minimum error value to avoid over-fitting.

Data Set IIIa is a four classes data set with a 60-channel EEG signal sampled at 250 Hz and recorded from 3 subjects. The class labels are *left hand*, *right hand*, *foot* and *tongue*. For each subject, there are minimum 60 trials per class; however, some of the trials are marked with *rejected trial*. Detailed information about the data sets may be found on the web site of the third BCI competition [25] and in the related paper of Blankertz et al. [11].

### Preprocessing, network configuration

**Fig 6:**
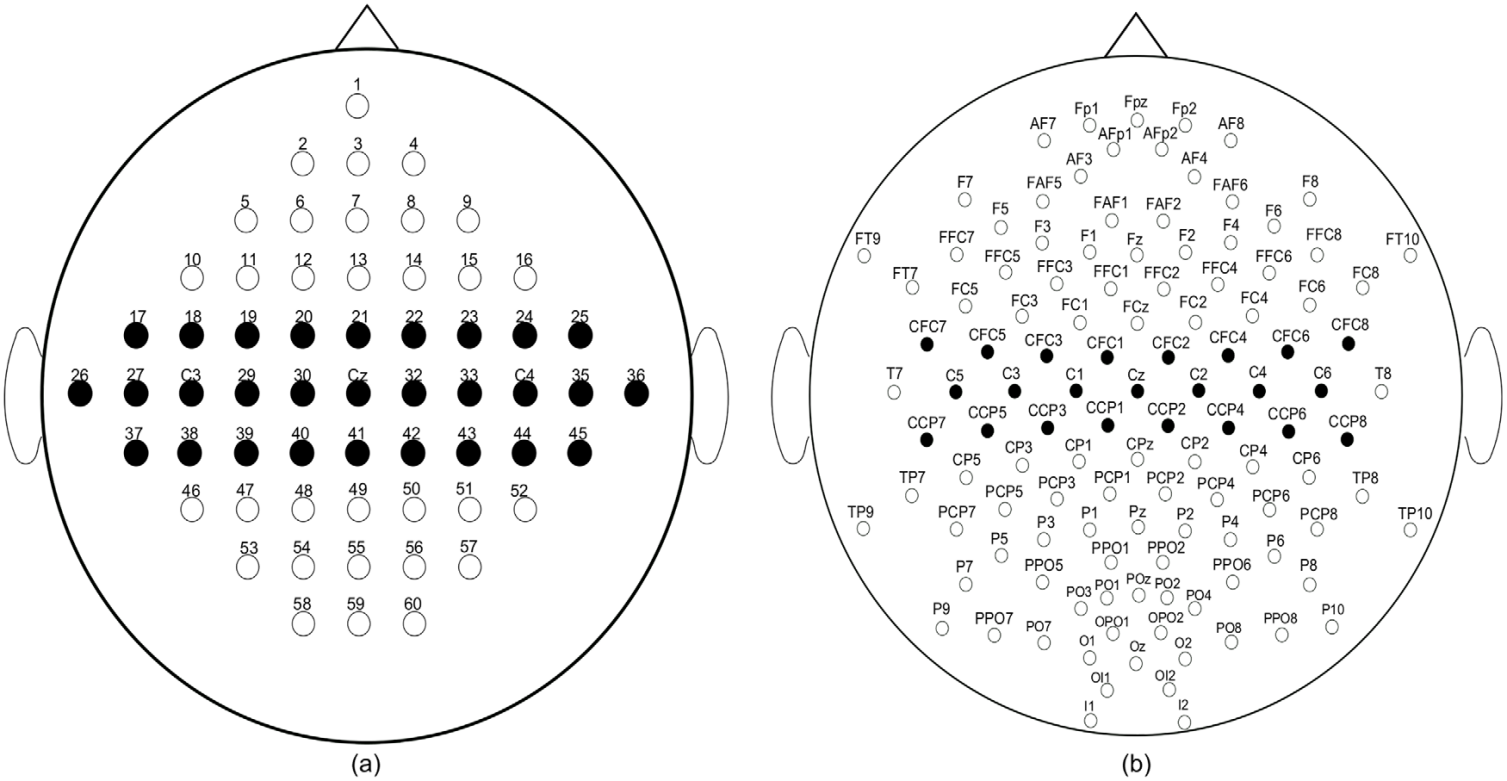
Selected electrodes (black circles) for BCI competition III Data Set IIIa (a) and BCI competition III Data Set IVa (b).

**Fig 7:**
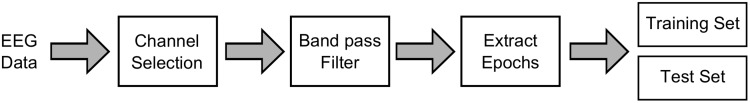
Preprocessing phase for EEG data sets.

We applied the same preprocessing steps for both data sets: i) We used EEG electrodes that roughly cover the motor cortex. Selected electrodes for the two data sets are shown in Fig 6. The numbers of electrodes are 23 and 29 for the the two classes data set (BCIC III-IVA) and for the four classes data set (BCIC III-IIIA), respectively. ii) The EEG signal is band bass filtered with a 8–30 Hz 5th-order Butterworth filter. iii) For each trial, we used EEG signals in the time segment between 0.5s–2s after the instruction cue. Furthermore, trials marked with *rejected trial* were excluded. The preprocessing phase is illustrated in Fig 7.

We used one-versus-rest CSP for comparison with SFN. As a classifier to classify the output of OVR-CSP, we selected linear discriminant analysis (LDA), which is a popular classifier in MI classification. OVR-CSP may be configured via setting the number of spatial filters for each class (*m*). In this study, we tested OVR-CSP with various values for *m* along with various spatial filter layer matrix widths (*M*) for SFN. We trained SFN with the LM method because of its convergence speed. SFN was configured as follows: *μ*_0_ = 100, *β* = 2, *EMIN* = 10^−1^ and *ITRMAX* = 1000.

## Results

**Fig 8:**
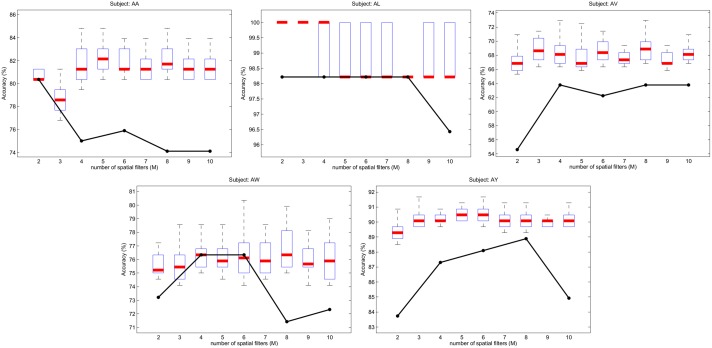
Accuracy of SFN and OVR-CSP over the subjects of data set BCIC-III-IVA.

**Fig 9:**
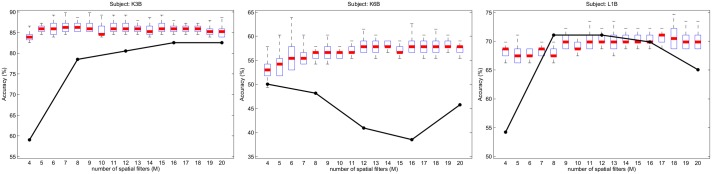
Accuracy of SFN and OVR-CSP over the subjects of data set BCIC-III-IIIA.

Figs 8 and 9 show the SFN accuracies for subjects of BCIC-III-IVA and BCIC-III-IIIA, respectively. For any subject and *m* value, the accuracy of OVR-CSP is constant for every running of the algorithm because OVR-CSP does not accept any hyper parameter and because it does not need an initial weight setting. However, because the initial weights were set randomly, the obtained accuracies vary at each running of the SFN. Therefore, SFN accuracies were plotted with box plots, where the box boundaries represent the upper and lower 25% quantiles of the input data (outliers were excluded), which was obtained by running the proposed algorithm 30 times for each user and *m* value. The bold lines inside the boxes represent the median values. Note that the total number of spatial filters (*M*) for OVR-CSP is a multiple of the number of classes. Therefore, the OVR-CSP method was run with *M* = [2, 4, 6, 8, 10] for the 2 classes data set BCIC-III-IVA and with *M* = [4, 8, 12, 16, 20] for the 4 classes data set BCIC-III-IIIA. The OVR-CSP accuracies for those *M* values are indicated with black dots in figures.

Although the classification accuracies vary significantly across subjects, it is clear that SFN increases the classification accuracy for most of the subjects in both data sets. For example, for subject *k6b*, whose classification results are the poorest in the 4 classes data set, SFN increases the accuracy by approximately 12%. Even though the proposed method may fall behind the multi-class CSP, it may achieve better accuracies in some trials (see the vertical lines above each box). Because the initial weights of SFN are set randomly, the accuracy for any subject and *M* value vary across each running of the algorithm. This situation may be improved with better and more robust selection of the initial network weights, which provides constantly higher results. However, increasing the robustness of the initial weight selection is beyond the scope of this paper.

## Discussion

### Spatial Filters

**Fig 10:**
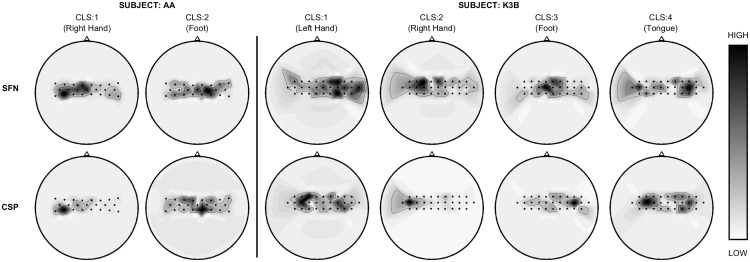
Illustration of spatial filters obtained with the SFN and CSP methods. Head figures are displayed for subject AA of data set BCIC-III-IVA (left) and subject K3B of dataset BCIC-III-IIIA. Each column corresponds to a class of the given data set.

The spatial filter layer of SFN aims to maximize the separability of the obtained features that belong to different classes. Thus, we expect each column of *W*, which is the weight matrix of SFN layer 1, to lighten a special area over the coverage of electrodes. We illustrate the spatial filters of the CSP and SFN methods in Fig 10 for 2 and 4 classes. Here, the upper and lower rows display the spatial filters of the SFN and CSP methods, respectively. Gray tones describe the absolute value of the illustrated spatial filter because the sign of the spatial filter weights does not affect the obtained energy features. Because the target functions of CSP and SFN are not identical, there are some differences between the the spatial filters obtained with these methods. However, both methods provide physiologically satisfying results. For example, right-hand motor imaginary is localized in the left hemisphere of the sensory-motor-cortex area, which is suitable with the Homunculus figure given in [26].

While CSP works with the average covariance matrices to calculate the spatial filters, SFN optimizes its spatial filters and classifier by handling each epoch in the training set. Therefore, spatial filters obtained with SFN provide *better* spatial filters that increase the between-class variance while decreasing within-class variance, thereby increasing the ratio of between-class variance and within-class variance, which is called the Fischer discriminant criterion or separation [27].

**Fig 11:**
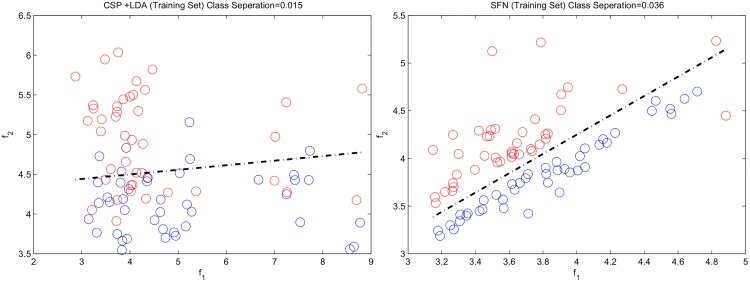
Features extracted with CSP (left) and SFN (right) from the training set of subject *av*. Blue: class 1, red: class 2. Dashed lines represents the class borders.

Fig 11 shows an example about separation of classes by using CSP and SFN spatial filters. Separation values for CSP and SFN features are given on the figure. As expected, SFN provides much higher separation value with proper organization of features (i.e. features belong to different classes separated in feature space) Class border given for CSP method was found by using LDA classifier while it was directly calculated for SFN algorithm.

### Convergence of network

**Fig 12:**
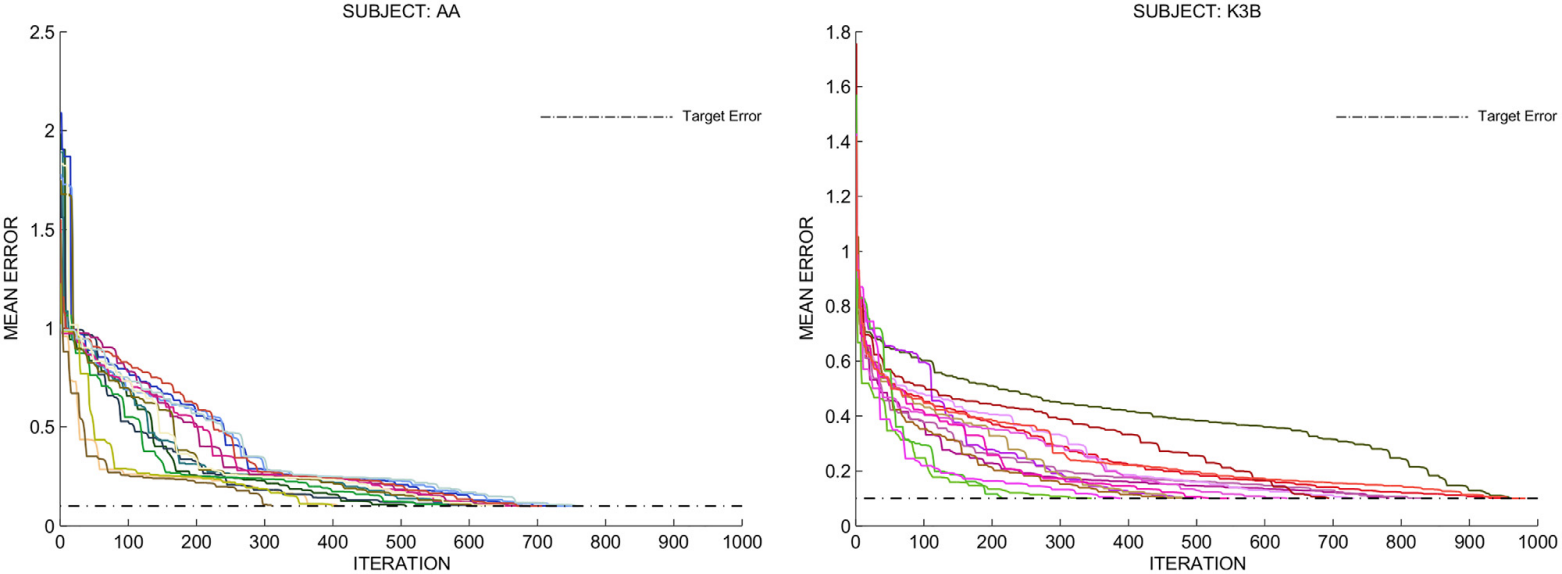
Converge behavior of the SFN. Network had been trained 15 times for subject AA (on the left) and for subject K3B (on the right). Resulting mean error value at each iteration until convergence for each training experiment is displayed in different colors.

We analyzed the convergence behavior of the network. As previously stated, the Levenberg-Marquardt (LM) method is used for training. Because the LM method has superior convergence properties, as proven in previous papers [28, 29], we expect SFN to converge to a desired error value. Fig 12 displays the mean error at each iteration for multiple training experiments with subjects AA and K3B. SFN reaches the desired error at each experiment for both of the subjects. However, the average number of iterations until convergence is higher for subject K3B of the data set BCIC-III-IIIA, which is a 4 classes data set.

### Training time

**Fig 13:**
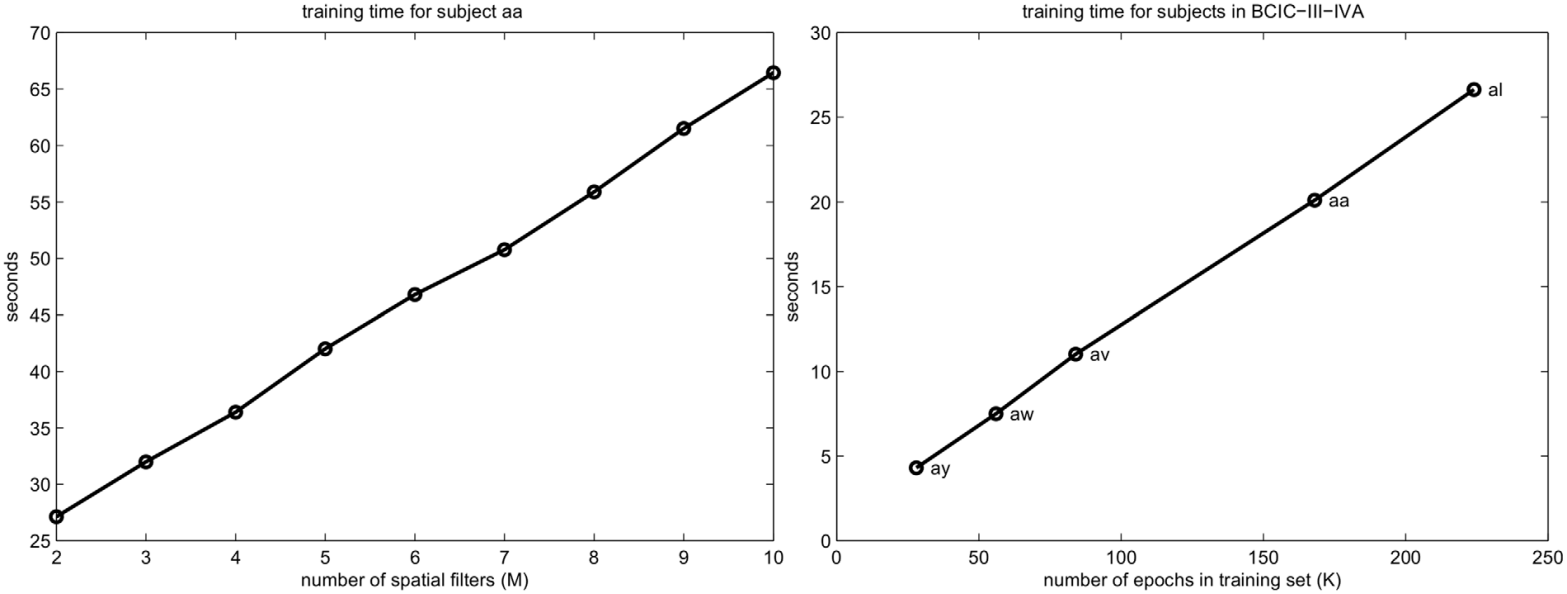
Training time of SFN. Left: number of spatial filters (*M*) versus training time for subject *aa*. Right: number of epochs versus training time for the subjects of BCIC-III-IVA when *M* = 2. SFN was configured with *EMIN* = 0, *ITRMAX* = 1000, *μ* = 100 and *B* = 2.

Because SFN learns the training set iteratively, the training time is longer than that for any direct method, such as CSP. Fig 13 plots the average training times for subject *aa* in the BCIC-III-IVA data set versus the number of spatial filters (*M*). This figure also plots the training time across each subject, where each subject has a different number of training sets. As shown in this figure, the required time for training the network increases linearly with the size of the SFN weight matrices and the number of epochs in the training set. The algorithm was run on a single-core Intel^®^ Xeon^®^ CPU operating at 2 GHz.

Although the training of SFN takes a long time, once the network is trained, the classification time for an epoch is very small. Therefore, a longer training time is not an obstacle for such a BCI system because training is performed off-line most of the time.

## Conclusions

In this study, we presented a combined method called spatial filter network (SFN) that combines spatial filter optimization and classification for motor imagery-based BCIs. The results indicated that the SFN method is an alternative approach to CSP with increased classification performance on two and four classes data sets and that it has two main advantages: ability to handle each single epoch in the training set and combining of spatial filter and classifier in a single structure. The originality of this paper is the combined optimization of spatial filters and classifier for single trial EEG motor imagery experiments. To the best of our knowledge, there are no methodologies in the literature that combine a spatial filter and classifier for such signals.

The following further work can be considered in future studies: searching for better initial weights to obtain consistently higher accuracies, studying more robust training methods that are insensitive to the outliers, and modifying the training algorithm for more efficient and faster convergence. Additionally, we could study the effect of increasing the number of spatial filter layers that are connected to each other with non-linear functions. To the best of our knowledge, multi-layer spatial filters have not previously been studied, and there may be some interesting results, particularly for dealing with non-stationary signals.

## Supporting Information

S1 Video An animation captured while training SFN for subject *av*. Number of spatial filters was set to *M* = 2 for display purposes. Left: Training set features ($\vec{f}\in {\mathbb{R}}^{2x1}$) scattered on two-dimensional feature space, where each circle represents an epoch that belongs to either class 1 (blue) or class 2 (red). Class separation value is displayed on the top of the window. Class border found by classifier layer is plotted with a black dashed line. Note that the two axes were normalized to the range [0–1] for display purposes. Right: Mean error of the current iteration was plotted with target error value (green horizontal line). Error value is displayed on the top of the window.
(MP4)

S1 Code Matlab code for SFN training and classification routines. See README and specific help texts of the supplied files.
(M) Click here for additional data file.
